# The monocyte to lymphocyte ratio not only at baseline but also at relapse predicts poor outcomes in patients with hepatocellular carcinoma receiving locoregional therapy

**DOI:** 10.1186/s12876-022-02180-6

**Published:** 2022-03-04

**Authors:** Qi Wang, Wenying Qiao, Biyu Liu, Jianjun Li, Chunwang Yuan, Jiang Long, Caixia Hu, Chaoran Zang, Jiasheng Zheng, Yonghong Zhang

**Affiliations:** 1 Research Center for Biomedical Resources, Beijing You’an Hospital, Capital Medical University, 8 Xitoutiao, Youanmenwai Street, Fengtai District, Beijing, 100069 China; 2 Interventional Therapy Center for Oncology, Beijing You’an Hospital, Capital Medical University, Beijing, 100069 China

**Keywords:** Hepatocellular carcinoma, Monocyte to lymphocyte ratio, Prognosis

## Abstract

**Background:**

Monocyte to lymphocyte ratio (MLR) represents a pro-inflammatory immune microenvironment. The aim of this study was to elucidate the effect of MLR and subsequent MLR when relapse occurred (R-MLR) on prognosis for hepatocellular carcinoma (HCC) after transcatheter arterial chemoembolization (TACE) combined with ablation.

**Methods:**

A prospective analysis was conducted on 606 patients with HCC who were treated with TACE combined with local ablation in Beijing You’an Hospital affiliated to Capital Medical University from January 1, 2012 to December 31, 2016. MLR or R-MLR were stratified according to the optimal cut-off values. The cumulative recurrence-free survival (RFS), overall survival (OS) , and recurrence-death survival (RDS) rates were calculated by Kaplan–Meier method. The Cox proportion hazard model and logistic regression analysis was conducted to screen for independent predictive factors for indicating early relapse and long-term prognosis.

**Results:**

High MLR was significantly associated with relapse, early recurrence, and overall survival. After a median follow-up of 59.4 months, The cumulative 1-, 3-, 5-year RFS rates of low MLR were 74.6%, 43.8%, and 34.0%; while 66.1%, 32.2%, and 22.6% for high group (*P* < 0.001). There were also significant differences in corresponding OS rates of the two groups (*P* = 0.003). The cumulative 1-, 3-, 5-year OS rates of low R-MLR were 99.5%, 87.2%, 75.5%; while 98.3%, 78.3%, 61.7% for high group (*P* < 0.001). There were also significant differences in corresponding RDS rates in the two groups (*P* = 0.008). 436 patients were divided into four groups on the base of cut-off values of MLR and R-MLR (low-low, low–high, high-low, and high-high). The low-low group has shown better outcomes including the cumulative 1-, 3-, 5-year OS, and RDS rates(*P* < 0.001).

**Conclusions:**

High MLR was related to unfavorable outcome. Subsequent change of MLR between baseline and HCC relapse could indicate poor long-term survival after relapse.

## Background

Hepatocellular carcinoma (HCC) as the most common type of liver cancer, is the third leading cause of cancer death worldwide [1]. HCC is also one of the few malignancies that is gradually increasing in both incidence and mortality in America [2]. More recently, Immune checkpoint inhibitors has been increasingly used to treat patients with advanced HCC [3–6]. However, the long-term prognosis of HCC is still awful and dismal on account of the low rate of early diagnosis, the heterogeneity of oncology, and the high rate of early recurrence [7].

It is well known that baseline values of neutrophil to lymphocyte ratio (NLR) and platelet to lymphocyte ratio (PLR) are predictors of undesirable prognosis in patients with HCC after treatments, such as liver resection or TACE [8–10]. However, no studies have demonstrated that monocyte to lymphocyte ratio (MLR) at baseline is associated with poor prognosis in HCC patients who receive local ablation, let alone to focus on the MLR at the time of recurrence (R-MLR).

We believe that inflammatory status during recurrence is a better indicator of tumor biological behavior and a better predictor of survival after recurrence. Therefore, a prospective cohort analysis with long-term follow-up has been performed to explore the prognostic value of baseline MLR, subsequent MLR, and MLR alteration in HCC patients who are treated with transcatheter arterial chemoembolization (TACE) combined with local ablation. Because all patients underwent ablation after TACE, more appropriate definition of “sequential therapy” was adopted hereafter [11].

## Material and methods

### Study subjects

A total of 606 HCC patients who were admitted to Beijing You 'an Hospital affiliated to Capital Medical University from January 1, 2012 to December 31, 2016 were included. The diagnosis of HCC is based on alpha fetoprotein (AFP), classic imaging features, and histological biopsy, which conforms to the suggestions of American Association for the Study of Liver Diseases (AASLD) [12]. Many literatures have confirmed that sequential therapy is superior to ablation or TACE alone, and this has been proved in our center, where the 5-year survival rate of HCC patients receiving the sequential therapy was 73%. Therefore, in order to improve the long-term prognosis, sequential therapy were performed in all patients that need to receive ablation or TACE alone recommended by guideline. All patients aged between 18 and 75 and treated with sequential therapy as the initial therapeutic schedule were enrolled. In order to avoid the effect of incomplete ablation as a confounding factor on poor prognosis, all patients recruited achieved complete ablation. Subjects must be excluded if they meet the following criteria: (1) had an infection or other inflammation when blood sampling; (2) Child–Pugh class C; (3) secondary liver cancer; (4) accompanied by other malignancies (other types of tumors or severe coagulopathy); (5) advanced stage of HCC.

Because people underwent resection or liver transplantation may suffer different prognosis, we enrolled patients who only received TACE or/and ablation as subsequent treatment modalities at the time of HCC recurrence.

The patients’ data were kept confidential. As a minimum risk study that was in accordance with the Helsinki protocol, the requirement for patients’ informed consent was waived, and it was approved by the ethics committee of Beijing You 'an Hospital.

### Data collection and presentation

The necessary data were collected within 7 days before treatment and at the time of diagnosis of the first recurrence, and were summarized as follows: (1) demographic information, like age, sex, history of hypertension and diabetes mellitus, history of smoking and drinking, history of antiviral therapy; (2) etiologies of HCC, such as hepatitis B virus (HBV), hepatitis C virus (HCV), co-infection, alcoholic liver disease (ALD) and others; (3) tumor-related data, including tumor number, tumor size (maximum diameter of tumor), alpha fetoprotein (AFP), and viral load; (4) liver function status, like cirrhosis, Child–Pugh class; (5) laboratory indicators, such as alanine aminotransferase (ALT), aspartate aminotransferase (AST), total bilirubin (TBIL), serum albumin, international normalized ratio (INR), NLR, PLR and MLR; and (6) ablation-related factors, including the ablation modality and in one-session or not. The NLR was computed as the absolute value of neutrophil divided by the lymphocyte; PLR was calculated as the platelet divided by the lymphocyte; and MLR was estimated as the absolute value of monocyte divided by the lymphocyte.

### Therapeutic procedure

All enrolled patients were treated with TACE combined with local ablation, which were performed by three qualified interventional radiologists. The modified Seldinger method was used to puncture the femoral artery. Microcatheter was inserted into the supply artery of the tumor, and doxorubicin (Pfizer Inc., NY, USA) coupled with lipiodol (Guerbet, Villepinte, France) were injected. Gelfoam were used to embolize the tumor supply artery, and its occlusions were considered as the complete embolization.

Ablation was carried out within 2 weeks after TACE. The whole ablation process was completed under computed tomography (CT) or magnetic resonance imaging (MRI) guidance. Overlapping ablation, multiple sites ablation, and repeated ablation were performed on the base of the number and size of tumors. In order to ensure complete coverage, a safety margin of 0.5–1.0 cm of the adjacent non-tumor tissue was ablated.

### Follow-up

Abdominal CT or MRI was performed about one month after ablation to identify whether complete ablation has been achieved. If not, a salvage treatment like TACE or ablation was carried out until the ablation was complete. The follow-up content included the physical examinations and blood test every 3 months, ultrasonography every 3–6 months, and CT/MRI every 6 months.

Recurrence would be confirmed when the imaging results showed an enhanced signal within, adjacent to, or out of the primary lesions. In order to avoid the variability of the evaluation of different radiologists, we set up the assessment team composed of three experienced radiologists with at least 5 years operating experience. And if there was any disagreement, it will be unanimously approved through group discussion. The recurrence-free survival (RFS) was described as the time span from the diagnosis to the first detectable recurrence or the date of death without HCC-related evidence. The overall survival (OS) was delineated as the time span from the diagnosis to HCC-related mortality or the last follow-up. The recurrence-death survival (RDS) was considered as the time span from the diagnosis of the first relapse to HCC-related mortality or the last follow-up. The last follow-up date was July 1, 2020. Individuals were treated with TACE or ablation therapies such as radiofrequency ablation (RFA), microwave ablation (MWA), and argon-helium knife cryoablation (AHC) when relapse was recognized.

### Statistical analysis

Continuous data, reported as the mean ± standard deviation, were performed by the Student’ t-test. Categorical data, expressed as the frequency, were compared by the Chi-square test. MLR or R-MLR were stratified according to the optimal cut-off values which were determined by Youden’s index. The Cox proportional hazard model was used to determine independent predictors of recurrence or long-term survival. The logistic regression analysis was used to determine independent parameters of early recurrence. The cumulative RFS, OS and RDS rates were calculated by Kaplan–Meier method and were compared by the log-rank test. *P* value < 0.05 was deemed to statistically significant. All the data were processed by IBM SPSS version 21.0 (SPSS Inc., Chicago, IL, USA) and R Foundation Statistical software (version 3.6.3).

## Results

### Baseline characteristics and follow-up results

The protocol included 490 (80.9%) males and 116 (19.1%) females, with an average age of 56 years old in diagnosis. 301 (49.7%) patients had the history of antiviral therapy. There were 446 (73.6%) cases in group of Child–Pugh class A and the rest in group of Child–Pugh class B. 500 (82.5%), 58 (9.6%), and 25 (4.1%) subjects were HBV-, HCV-, and co-infection-related HCC, while 15 (2.5%) and 8 (1.3%) were associated with ALD and others, respectively. There were 500 (82.5%) individuals with cirrhosis. 312 (51.5%), 157 (25.9%), and 137 (22.6%) patients were treated with RFA, MWA and AHC, respectively. 523 (86.3%) cases received ablation in only one session while others were administrated fractionally.

AFP values were missing in 4 cases and viral load were missing in 43 cases, but the two were included in the analysis as they were only a small part of it. The median OS was not achieved, while the median RFS was 24.5 months. By the end of follow-up, a total of 446 (73.6%) patients were diagnosed with recurrence and 212 (35.0%) died of HCC with a median follow-up of 59.4 months (25–75th percentiles, 44.3–81.0 months). Of the patients who relapsed, 303 (67.9%) had an early recurrence within two years after discharge. The cumulative recurrence-free survival (RFS) rates of 1, 3, and 5 years were 70.0% (424/606), 40.1% (243/606), and 30.5%, and the corresponding OS rates were 99.0% (600/606), 85.5% (518/606), and 73.3% (444/606), respectively.

### Prognostic factors related to RFS, OS, and early recurrence

Due to frequent recurrence and subsequent poor prognosis, we screened for independent risk factors of predicting RFS and OS by the Cox proportional hazards regression analysis (Tables 1 and 2). The univariate and multivariate analysis identified that old age, sex, cirrhosis, multiple tumors, larger tumors, AST, and high MLR were in connection with the higher recurrence rates. Some parameters including old age, the history of drinking, Child–Pugh lass B, multiple tumors, and high MLR indicated the inferior OS in HCC patients received sequential therapies.

**Table 1:** Prognostic factors associated with RFS by Cox proportional hazards regression model

Varibles	Univariate		Multivariate	
	HR (95% CI)	*P* value	HR (95% CI)	*P* value
Age (years old)	1.02 (1.00–1.03)	**0.006**	1.02 (1.00–1.03)	**0.008**
Sex (male/female)	1.61 (1.25–2.08)	** < 0.001**	1.70 (1.30–2.22)	** < 0.001**
Hypertension (yes/no)	0.99 (0.79–1.23)	0.907		
Diabetes mellitus (yes/no)	1.08 (0.85–1.37)	0.545		
Smoking history (yes/no)	1.17 (0.97–1.41)	0.094		
Drinking history (yes/no)	1.14 (0.94–1.38)	0.202		
Antivival history (yes/no)	0.87 (0.72–1.05)	0.151		
Etiology (HBV/HCV/Co-infection/others)	0.99 (0.90–1.09)	0.791		
Cirrhosis (yes/no)	1.36 (1.04–1.77)	**0.023**	1.36 (1.02–1.81)	**0.036**
Child–Pugh class (A/B)	1.20 (0.96–1.48)	0.084		
Fractional ablation (yes/no)	1.52 (1.18–1.97)	**0.001**	0.92 (0.68–1.24)	0.571
Ablative modality (RFA/MWA/AHC)	0.94 (0.84–1.05)	0.300		
Tumor number (single/multiple)	1.58 (1.30–1.91)	** < 0.001**	1.61 (1.30–1.99)	** < 0.001**
Tumor size (≤ 30 mm/ > 30 mm)	1.74 (1.43–2.11)	** < 0.001**	1.61 (1.29–2.01)	** < 0.001**
Alanine aminotransferase (U/L)	1.00 (0.99–1.01)	0.302		
Aspartate aminotransferase (U/L)	1.01 (1.00–1.01)	**0.001**	1.01 (1.00–1.01)	**0.012**
Total bilirubin (μmol/L)	1.01 (1.00–1.02)	**0.023**	1.00 (0.99–1.01)	0.691
Albumin (g/l)	0.96 (0.94–0.98)	** < 0.001**	0.99 (0.96–1.02)	0.437
INR	2.37 (1.14–4.91)	**0.021**	1.16 (0.41–3.30)	0.779
Alpha fetoprotein (< 7/7–400/ > 400 ng/mL)	1.22 (1.06–1.41)	**0.007**	1.15 (0.98–1.34)	0.091
Viral load (< 1000/1000–20,000/ > 20000 IU/mL)	1.14 (1.02–1.28)	**0.019**	1.03 (0.91–1.16)	0.648
NLR	1.03 (0.98–1.06)	0.077		
PLR	1.00 (1.00–1.00)	0.109		
MLR	1.99 (1.36–2.91)	** < 0.001**	1.65 (1.08–2.53)	**0.022**

Bold values mean that the *P* value is less than 0.05, and there is statistical difference

HBV: hepatitis B virus; HCV: hepatitis C virus; ALD: alcoholic liver disease; RFA: radiofrequency ablation; MWA: microwave ablation; AHC: argon-helium knife cryoablation (AHC); INR: international normalized ratio; NLR: the neutrophil to lymphocyte ratio; PLR: the platelet to lymphocyte ratio; MLR: the monocyte to lymphocyte ratio

**Table 2:** Prognostic factors associated with OS by Cox proportional hazards regression model

Varibles	Univariate		Multivariate	
	HR (95% CI)	*P* value	HR(95% CI)	*P* value
Age (years old)	1.02 (1.01–1.04)	**0.005**	1.03 (1.01–1.04)	**0.005**
Sex (male/female)	1.56 (1.06–2.30)	**0.024**	1.18 (0.75–1.84)	0.481
Hypertension (yes/no)	0.88 (0.63–1.22)	0.430		
Diabetes mellitus (yes/no)	1.11 (0.79–1.57)	0.553		
Smoking history (yes/no)	1.41 (1.08–1.85)	**0.012**	1.22 (0.88–1.68)	0.230
Drinking history (yes/no)	1.64 (1.25–2.15)	** < 0.001**	1.61 (1.17–2.22)	**0.004**
Antivival history (yes/no)	0.73 (0.56–0.96)	**0.024**	0.91 (0.67–1.25)	0.574
Etiology (HBV/HCV/co-infection/others)	1.17 (1.04–1.32)	**0.008**	1.13 (0.98–1.30)	0.103
Cirrhosis (yes/no)	1.28 (0.85–1.91)	0.234		
Child–Pugh class (A/B)	1.88 (1.42–2.50)	** < 0.001**	1.59 (1.11–2.29)	**0.012**
Fractional ablation (yes/no)	1.53 (1.08–2.17)	**0.018**	1.09 (0.73–1.64)	0.668
Ablative.modality (RFA/MWA/AHC)	1.08 (0.92–1.27)	0.359		
Tumor number (single/multiple)	1.54 (1.17–2.02)	**0.002**	1.48 (1.10–2.00)	**0.011**
Tumor size (≤ 30 mm/ > 30 mm)	1.38 (1.04–1.83)	**0.026**	1.19 (0.85–1.66)	0.307
Alanine aminotransferase (U/L)	1.00 (1.00–1.01)	0.230		
Aspartate aminotransferase (U/L)	1.01 (1.00–1.01)	**0.007**	1.01 (1.00–1.01)	0.146
Total bilirubin (μmol/L)	1.02 (1.01–1.03)	**0.004**	0.99 (0.98–1.01)	0.330
Albumin (g/l)	0.93 (0.90–0.96)	** < 0.001**	0.98 (0.95–1.03)	0.440
INR	6.97 (2.62–18.54)	** < 0.001**	3.33 (0.80–13.92)	0.100
Alpha fetoprotein (< 7/7–400/ > 400 ng/mL)	1.09 (0.88–1.350	0.407		
Viral load (< 1000/1000–20,000/ > 20000 IU/mL)	1.23 (1.05–1.44)	**0.012**	1.10 (0.92–1.33)	0.295
NLR	1.02 (0.98–1.06)	0.411		
PLR	1.00 (1.00–1.00)	0.130		
MLR	2.05 (1.21–3.45)	**0.007**	1.95 (1.07–3.57)	**0.029**

Bold values mean that the *P* value is less than 0.05, and there is statistical difference

HBV: hepatitis B virus; HCV: hepatitis C virus; ALD: alcoholic liver disease; RFA: radiofrequency ablation; MWA: microwave ablation; AHC: argon-helium knife cryoablation (AHC); INR: international normalized ratio; NLR: the neutrophil to lymphocyte ratio; PLR: the platelet to lymphocyte ratio; MLR: the monocyte to lymphocyte ratio

We continued to investigate whether MLR is associated with early recurrence by using logistic regression analysis. As expected, parameters including sex, cirrhosis, multiple tumors, large tumors, and high MLR had great predictive performance for early relapse (Table 3). Compared to patients with low MLR at baseline, those with high MLR had a 194% increased risk of early recurrence and a 95% increased risk of death.

**Table 3:** Prognostic factors related to early recurrence by logistic regression analysis

Varibles	Univariate		Multivariate	
	OR (95% CI)	*P* value	OR (95% CI)	*P* value
Age (years old)	1.01 (0.99–1.03)	0.206		
Sex (male/female)	1.84 (1.21–2.78)	**0.004**	1.68 (1.07–2.65)	**0.024**
Hypertension (yes/no)	0.91 (0.63–1.33)	0.636		
Diabetes mellitus (yes/no)	1.10 (0.72–1.66)	0.671		
Smoking history (yes/no)	1.36 (0.99–1.88)	0.060		
Drinking history (yes/no)	1.26 (0.90–1.77)	0.171		
Antivival history (yes/no)	0.73 (0.52–1.00)	0.051		
Etiology (HBV/HCV/Co-infection/others)	0.95 (0.78–1.15)	0.582		
Cirrhosis (yes/no)	1.85 (1.19–2.88)	**0.007**	1.68 (1.01–2.79)	**0.047**
Child–Pugh class (A/B)	1.46 (1.01–2.10)	**0.043**	1.08 (0.67–1.74)	0.749
Fractional ablation (yes/no)	2.18 (1.34–3.54)	**0.002**	1.07 (0.61–1.88)	0.814
Ablative.modality (RFA/MWA/AHC)	0.95 (0.78–1.15)	0.582		
Tumor number (single/multiple)	2.08 (1.47–2.94)	** < 0.001**	1.99 (1.34–2.96)	**0.001**
Tumor size (≤ 30 mm/ > 30 mm)	2.36 (1.65–3.37)	** < 0.001**	2.22 (1.47–3.36)	** < 0.001**
Alanine aminotransferase (U/L)	1.01 (1.00–1.01)	0.096		
Aspartate aminotransferase (U/L)	1.02 (1.01–1.02)	**0.002**	1.01 (1.00–1.02)	0.064
Total bilirubin (μmol/L)	1.02 (1.01–1.04)	**0.005**	1.01 (0.98–1.03)	0.576
Albumin (g/l)	0.94 (0.91–0.98)	**0.001**	1.00 (0.95–1.04)	0.827
INR	3.83 (1.02–14.41)	**0.047**	1.07 (0.17–6.67)	0.945
Alpha fetoprotein (< 7/7–400/ > 400 ng/mL)	1.45 (1.12–1.88)	**0.005**	1.20 (0.90–1.62)	0.221
Viral load (< 1000/1000–20,000/ > 20000 IU/mL)	1.28 (1.05–1.57)	**0.015**	1.10 (0.87–1.34)	0.427
MLR	3.93 (1.82–8.49)	** < 0.001**	2.94 (1.28–6.77)	**0.011**

Bold values mean that the *P* value is less than 0.05, and there is statistical difference

HBV: hepatitis B virus; HCV: hepatitis C virus; ALD: alcoholic liver disease; RFA: radiofrequency ablation; MWA: microwave ablation; AHC: argon-helium knife cryoablation (AHC); INR: international normalized ratio; NLR: the neutrophil to lymphocyte ratio; PLR: the platelet to lymphocyte ratio; MLR: the monocyte to lymphocyte ratio

### The MLR-based analysis of clinicopathological and prognosis data

The cut-off value of MLR was defined as 0.44 in the present study based on recurrence. The MLR-based comparison of clinical data at baseline was shown in Table 4. High MLR was associated with large tumors, high ALT, high TBIL, low albumin, high INR, high NLR, and high PLR (*P* < 0.05). The number of relapse, early recurrence and mortality were significantly higher in the high MLR group than in the low MLR group with p values less than 0.05 (82.5%, 59.9%, 45.2% vs 69.9%, 45.9%, 30.8%).

**Table 4:** The MLR-based comparison of baseline clinical data

Variables	Total	MLR < 0.44	MLR ≥ 0.44	*P *value
		**n = 429**	**n = 177**	
Recurrence number	446	300	146	**0.001**
Early recurrence	303	197	106	**0.002**
Death number	212	132	80	**0.001**
Age (years old)	56.45 ± 8.82	56.35 ± 8.82	56.69 ± 8.83	0.669
Sex (male/female)	490/116	344/85	146/31	0.513
Hypertension (yes/no)	147/459	112/317	35/142	0.098
Diabetes mellitus (yes/no)	108/498	81/348	27/150	0.289
Smoking history (yes/no)	267/339	189/240	78/99	0.998
Drinking history (yes/no)	208/398	155/274	53/124	0.145
Antivival history (yes/no)	301/301	218/207	83/94	0.325
Etiology (HBV/HCV/co-infection/ALD/others)	500/58/25/15/8	348/44/19/12/6	152/14/6/3/2	0.722
Cirrhosis (yes/no)	507/99	359/70	148/29	0.984
Child–Pugh class (A/B)	446/160	316/113	130/47	0.957
Fractional ablation (yes/no)	83/523	56/373	27/150	0.474
Ablative modality (RFA/MWA/AHC)	312/157/137	219/103/107	93/54/30	0.060
Tumor number (single/multiple)	408/198	296/133	112/65	0.172
Tumor size (≤ 30 mm/ > 30 mm)	420/186	310/119	110/67	**0.014**
Alanine aminotransferase (U/L)	39.77 ± 26.76	37.70 ± 25.83	44.80 ± 28.34	**0.004**
Aspartate aminotransferase (U/L)	36.62 ± 19.66	36.07 ± 19.29	37.96 ± 20.55	0.283
Total bilirubin (μmol/L)	19.24 ± 10.17	17.81 ± 9.54	22.70 ± 10.84	** < 0.001**
Albumin (g/l)	37.03 ± 4.48	37.45 ± 4.54	35.99 ± 4.17	** < 0.001**
INR	1.08 ± 0.12	1.07 ± 0.12	1.10 ± 0.12	**0.023**
Alpha fetoprotein (< 7/7–400/ > 400 ng/mL)	251/299/52	186/204/37	65/95/15	0.319
Viral load (< 1000/1000–20,000/ > 20000 IU/mL)	308/118/137	224/83/91	84/35/46	0.401
NLR	3.23 ± 2.81	2.37 ± 1.78	5.30 ± 3.65	** < 0.001**
PLR	107.59 ± 54.03	96.55 ± 42.45	134.37 ± 68.09	** < 0.001**

Bold values mean that the *P* value is less than 0.05, and there is statistical difference

HBV: hepatitis B virus; HCV: hepatitis C virus; ALD: alcoholic liver disease; RFA: radiofrequency ablation; MWA: microwave ablation; AHC: argon-helium knife cryoablation (AHC); INR: international normalized ratio; NLR: the neutrophil to lymphocyte ratio; PLR: the platelet to lymphocyte ratio; MLR: the monocyte to lymphocyte ratio

**Fig. 1:**
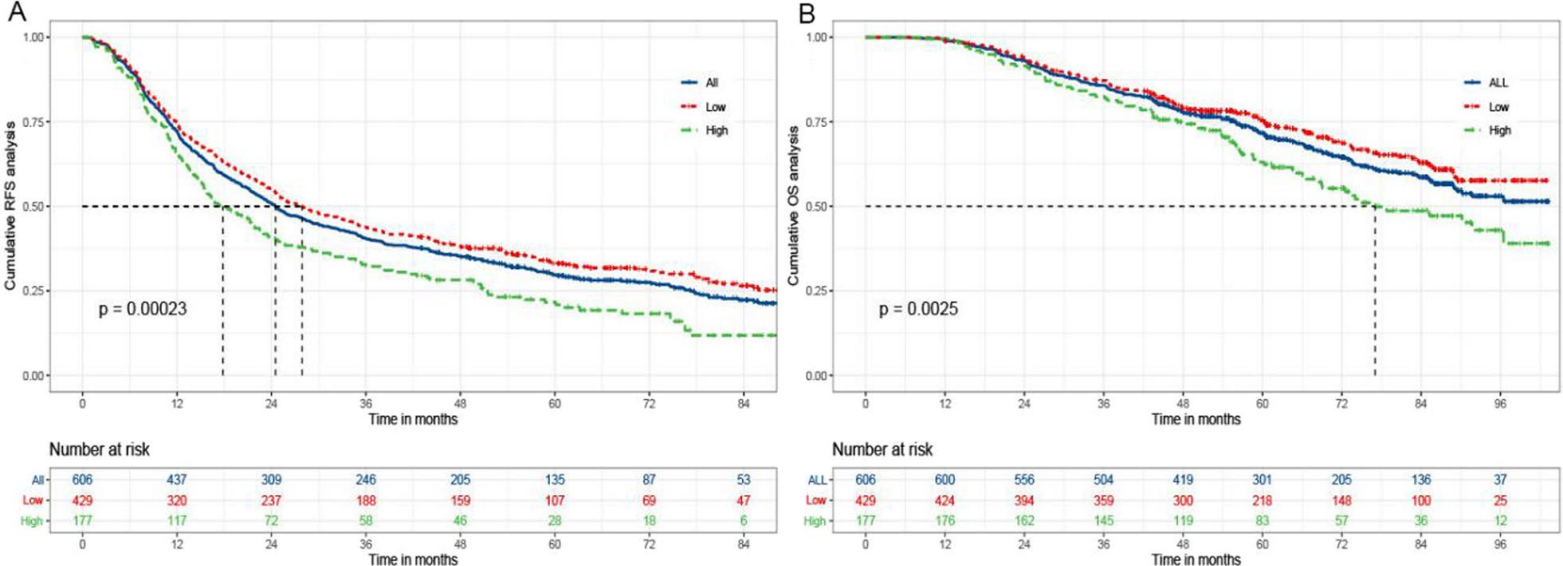
The Kaplan–Meier analysis of RFS and OS for patients with low and high MLR. RFS: Recurrence-free survival; OS: Overall survival; MLR: The monocyte to lymphocyte ratio. **a** Is the Kaplan–Meier analysis of RFS. **B** is the Kaplan–Meier analysis of OS

**Fig. 2:**
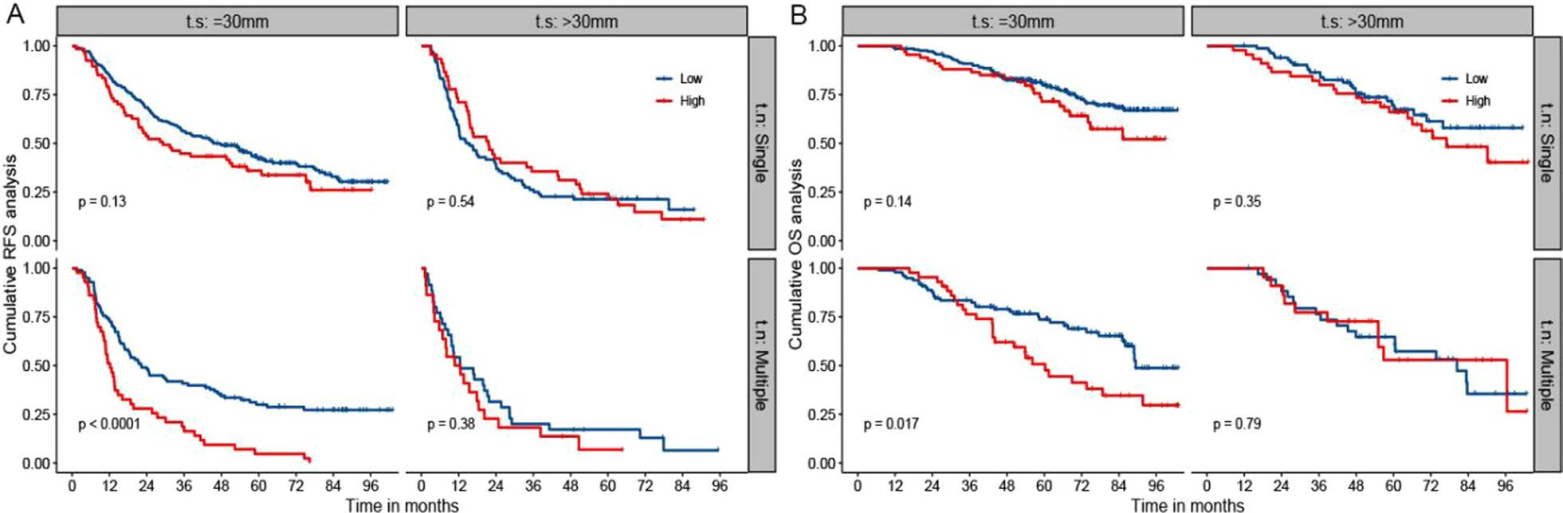
The Kaplan–Meier analysis of RFS and OS for patients based on MLR, tumor number, and tumor size. **a** is the Kaplan–Meier analysis of RFS; **b** is the Kaplan–Meier analysis of OS. RFS: recurrence-free survival; OS: overall survival; MLR: the monocyte to lymphocyte ratio. t.n: tumor number; t.s: tumor size

Until the end of follow-up, the median OS was not achieved in the low MLR group, while it was 77.0 months in the high MLR group. The median RFS of the two groups were 27.9 months and 17.8 months, respectively. The cumulative 1-, 3-, 5-year RFS rates of the former were 74.6% (320/429), 43.8% (188/429), and 34.0% (146/429); while 66.1% (117/177), 32.2% (57/177), and 22.6% (40/177) for the latter (*P* < 0.001). There were also significant differences in corresponding OS rates in the low and high MLR groups (98.8%, 87.4%, 77.2% vs 99.4%, 82.5%, 65.5%, *P* = 0.003) (Fig. 1). A subgroup analysis was performed to explore whether the MLR-based difference of prognosis was still significant in some populations stratified by tumor number and size. The results has shown that there were statistically significant differences in RFS and OS among patients with multiple, small tumors (Fig. 2).

### The analysis of prognosis data base on R-MLR

**Fig. 3:**
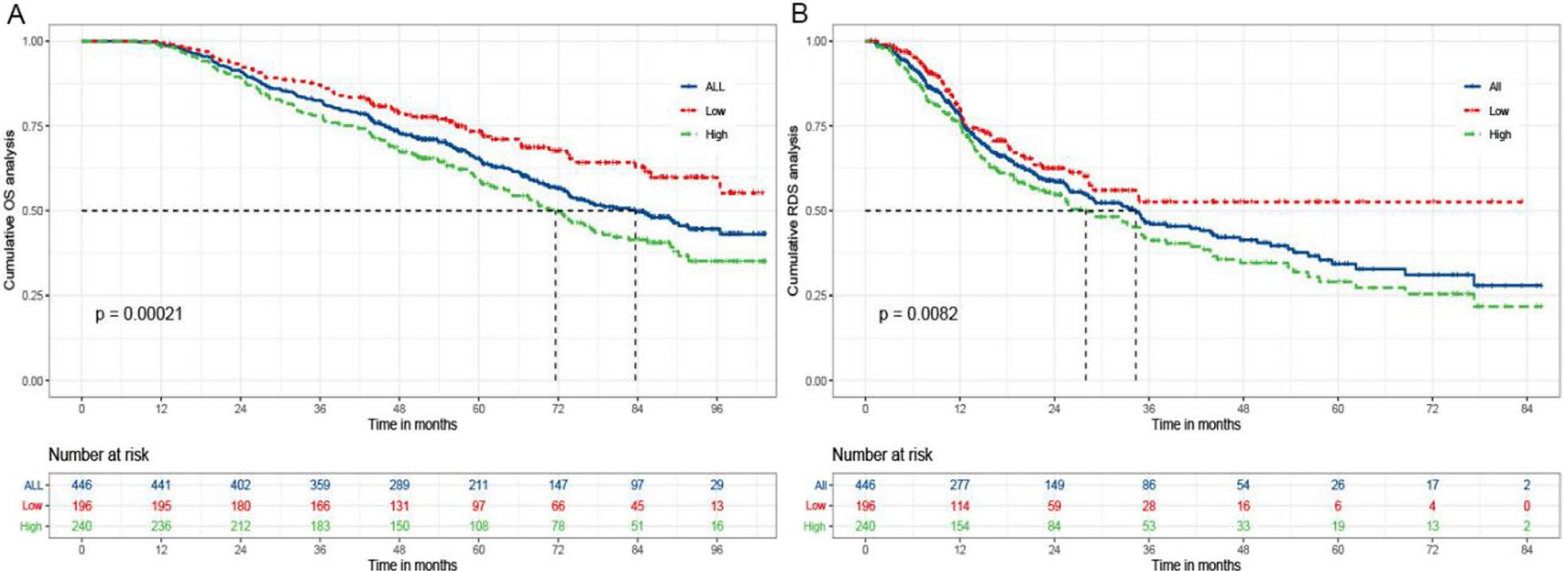
The Kaplan–Meier analysis of OS and RDS for patients with low and high R-MLR. **a** is the Kaplan–Meier analysis of OS; **b** is the Kaplan–Meier analysis of RDS. OS: overall survival; RDS: recurrence-death survival; MLR: the monocyte to lymphocyte ratio

**Fig. 4:**
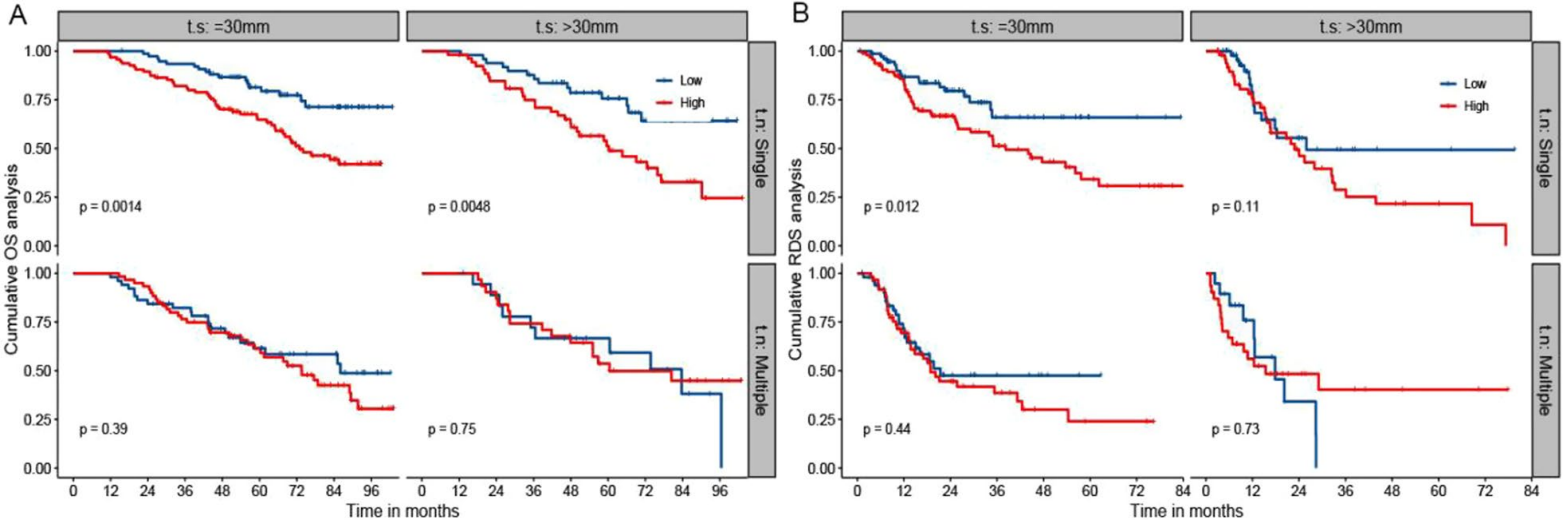
The Kaplan–Meier analysis of OS and RDS for patients based on R-MLR, tumor number, and tumor size. **a** is the Kaplan–Meier analysis of OS; **b** is the Kaplan–Meier analysis of RDS. OS: overall survival; RDS: recurrence-death survival; MLR: the monocyte to lymphocyte ratio. t.n: tumor number; t.s: tumor size

To clarify whether R-MLR at the time of relapse could also predict survival status after recurrence, we collected available MLR values for 436 patients when recurrence. The cut-off value of R-MLR was defined as 0.24 in the present study based on OS. The median OS and RDS was not achieved in the low R-MLR group, while it was 71.6 and 28.0 months in the high R-MLR group, respectively. There were 31.6% (62/196) deaths in low R-MLR group, while 52.5% (126/240) in high R-MLR group, respectively (*P* < 0.001). The cumulative 1-, 3-, 5-year OS rates of the former were 99.5% (195/196), 87.2% (171/196), 75.5% (148/196); while 98.3% (236/240), 78.3% (188/240), 61.7% (148/240) for the latter (*P* < 0.001). There were also significant differences in corresponding RDS rates in the two groups (83.2%, 68.4%, 68.4% vs 77.1%, 53.8%, 48.8%, *P* = 0.008) (Fig. 3). In the same way, a subgroup analysis was performed to explore whether the R-MLR-based difference of prognosis was still significant in some populations stratified by tumor number and size. As shown in the Fig. 4, there were significant differences in OS and RDS among patients with single tumor.

### The analysis of prognosis data base on MLR alteration

**Fig. 5:**
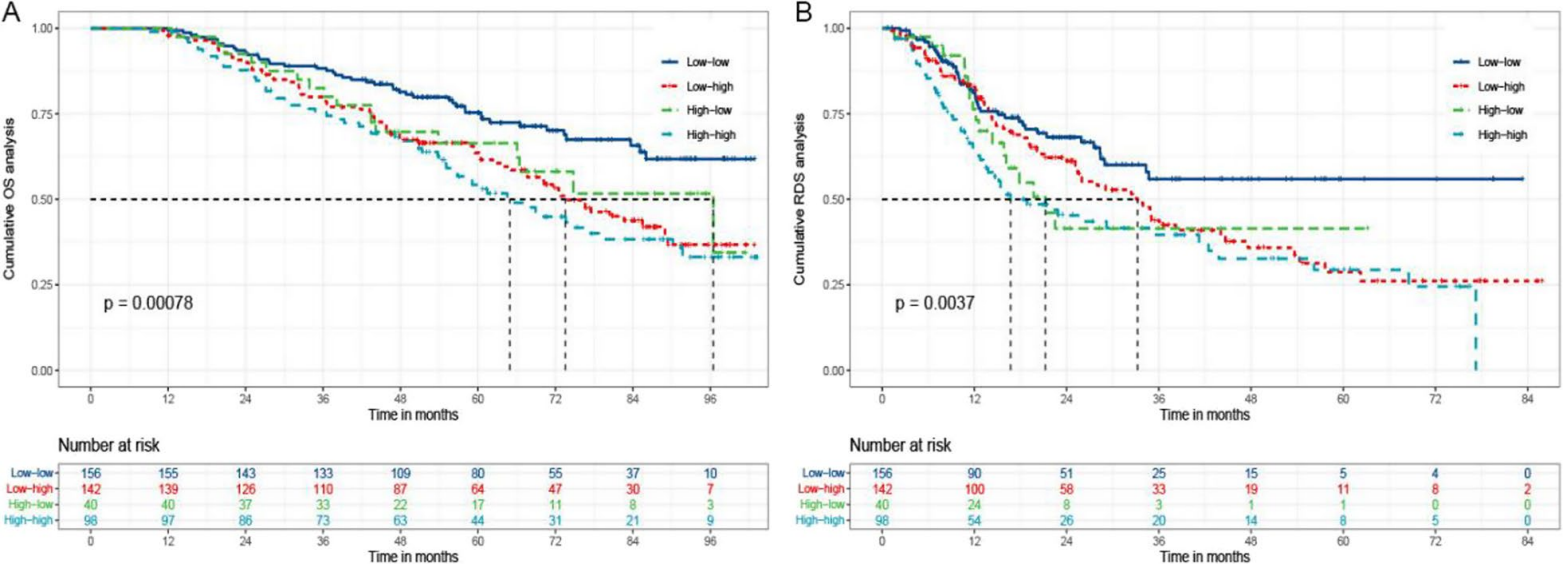
The Kaplan–Meier analysis of OS and RDS for patients based on MLR alteration. **a** is the Kaplan–Meier analysis of OS; **b** is the Kaplan–Meier analysis of RDS. OS: overall survival; RDS: recurrence-death survival; MLR: the monocyte to lymphocyte ratio

To unravel the relationship between MLR alteration and long-term survival, the patients were categorized according to MLR at baseline and tumor recurrence. 436 patients were divided into four groups on the base of cut-off values of MLR and R-MLR (low-low, low–high, high-low, and high-high). There were 28.8% (45/456), 49.3% (70/142), 42.5% (17/40), and 57.1% (56/98) deaths in low-low, low–high, high-low, and high-high groups (*P* < 0.001). The median OS and RDS was not achieved in the low-low group. The median OS and RDS of the other three groups were 73.5, 96.5, 64.9 and 33.2, 21.2, 16.7 months. The cumulative 1-, 3-, 5-year OS rates were 99.4% (155/156), 85.5% (138/156), and 77.6% (121/156) for patients in low-low group, 97.9% (139/142), 80.3% (114/142), and 65.5% (93/142) in low–high group, 100.0% (40/40), 82.5% (33/40), and 67.5% (27/40) in high-low group, and 99.0% (97/98), 75.5% (74/98), and 56.1% (55/98) in high-high group (*P* < 0.001). There were also significant differences in corresponding RDS rates in the four groups (84.0%, 71.2%, 71.2% vs 83.8%, 57.0%, 51.4% vs 80.0%, 57.5%, 57.5% vs 67.3%, 49.0%, 44.9%, *P* = 0.004) (Fig. 5). Patients in low-low group had longer OS and RDS than others. However, unfavorable long-term outcomes were noted for HCC patients with a high baseline or subsequent MLR at the time of recurrence.

## Discussion

The treatment model combined TACE with ablative therapy which was performed more and more widely, has obvious therapeutic effect in the field of HCC. Many studies have confirmed that patients receiving sequential therapy have longer OS and better prognosis than those only receiving TACE or ablative treatment [13, 14]. Our results have indicated that only 212 patients died by the end of follow-up. TACE could mark tumors that are not shown clearly on imaging, degrade the tumor by embolizing tumor blood vessels, reduce ablation times, and increase the success rate of ablation [15–17]. In contrast, ablation as a radical treatment could contribute to a better tumor response, which leads to reduced formulation of tumor collateral circulation [18]. It is hoped that the efficacy of sequential therapy will be validated in multiple centers and become a standard treatment for HCC, offering chances of survival in patients with intermediate- or advanced-staged HCC. Our results has shown that there was no difference in outcomes with respect to times and modalities of ablation. Therefore, if the tumor lesion could not be ablated completely in one session, fractional RFA, MWA could be considered [19].

Chronic inflammation has been shown to be associated with the development and progression of a variety of tumors, including HCC [20, 21]. Systemic inflammation not only influences the tumors’ immune microenvironment, but also impairs the nutritional status, which further increases the recurrence rate and mortality of HCC patients [22]. Therefore, some studies have confirmed that parameters representing systemic inflammation, such as NLR and PLR, can predict the prognosis of patients with HCC after treatment [23–25]. However, these studies have mostly been focused on patients who received liver resection and transplantation, or who have advanced liver cancer, and that's probably why NLR and PLR didn't make sense in our results [8–10, 26]. No one has shown that MLR can be associated with the prognosis of HCC patients who receive local ablation. Hence, for the first time, we have demonstrated the prognostic value of MLR including early recurrence and survival after relapse with a prospective cohort.

The mechanisms between MLR and prognosis remain unclear at present. Our study has revealed that MLR or subsequent R-MLR is a great predictor of prognosis, which might result from association of MLR with tumor aggressiveness and poor liver function in HCC patients. MLR is the ratio of monocyte to lymphocyte. Monocyte gathers at the inflammatory site and differentiates into M1 and M2 macrophages when inflammation occurs. The M2 macrophages have been reported to promote the development and progression of tumor [27]. Macrophages can promote angiogenesis and invasion by secreting vascular endothelial growth factor (VEGF) [28]. The chitinase 3-like protein 1 (CHI3L1) secreted by M2 macrophages specifically binds to the interleukin (IL)-13 receptor α2 chain (IL-13Rα2) of gastric and breast cancer cells, which contributes to tumor metastasis [29]. In addition, macrophages inhibit the anti-tumor effect of CD4 + T cells, and inhibit T cell proliferation by secreting several molecules like TGF-β and Arg-1 [30].

A negative correlation between lymphocytes and tumor stage has been reported [31]. Lymphocytes which reflects the immune response status, play a key role in the anti-tumor response. Therefore, MLR may be a potentially negative prognostic marker for HCC patients. In this cohort, the median RFS in patients with elevated MLR was significantly lower than those with low MLR (17.8 months vs. 27.9 months, *P* < 0.001), which is consistent with Wu’s conclusions [32].

Although this is a single-center study, it is the first to validate the prognostic value of MLR, R-MLR, and MLR alteration in HCC patients receiving local treatment with a large sample size and a prospective cohort. The present study has a profound guiding significance for clinical practice. The physicians should adjust follow-up strategy or take timely intervention measures to improve the outcomes of patients with high baseline and/or subsequent R-MLR at recurrence.

## Conclusions

In conclusion, the present study was conducted based on a large sample of HCC patients who undergone TACE combined with ablation and verified that high MLR was an independent unfavorable predictor of early recurrence and overall survival, and that similar predictive value of subsequent R-MLR was also confirmed. The MLR alteration between baseline and HCC relapse could predict long-term survival after recurrence.

## Data Availability

The data used to support the findings are available from the corresponding author upon request.

## Abbreviations

MLR: Monocyte to lymphocyte ratio
R-MLR: MLR when relapse
HCC: Hepatocellular carcinoma
TACE: Transcatheter arterial chemoembolization
RFS: Recurrence-free survival
OS: Overall survival
RDS: Recurrence-death survival
NLR: Neutrophil to lymphocyte ratio
PLR: Platelet to lymphocyte ratio
AFP: Alpha fetoprotein
AASLD: American association for the study of liver diseases
HBV: Hepatitis B virus
HCV: Hepatitis C virus
ALD: Alcoholic liver disease
ALT: Alanine aminotransferase
AST: Aspartate aminotransferase
TBIL: Total bilirubin
INR: International normalized ratio
CT: Computed tomography
MRI: Magnetic resonance imaging
RFA: Radiofrequency ablation
MWA: Microwave ablation
AHC: Argon-helium knife cryoablation
VEGF: Vascular endothelial growth factor
CHI3L1: Chitinase 3-like protein 1
IL-13Rα2: Interleukin (IL)-13 receptor α2 chain
